# Excellent clinical and radiological outcome following locking compression plate fixation of displaced medial clavicle fractures

**DOI:** 10.1186/s12891-021-04775-8

**Published:** 2021-10-27

**Authors:** Markus Wurm, Sebastian Siebenlist, Michael Zyskowski, Patrick Pflüger, Peter Biberthaler, Marc Beirer, Chlodwig Kirchhoff

**Affiliations:** 1 Department of Trauma Surgery, Klinikum rechts der Isar, Technical University Munich, Munich, Germany; 2 Department of Orthopaedic Sports Medicine, Klinikum rechts der Isar, Technical University Munich, Munich, Germany

**Keywords:** Displaced, Medial, Clavicle, Fracture, LCP, ORIF

## Abstract

**Background:**

Treatment of medial clavicle fractures is still controversially discussed in the community of upper extremity surgeons. An increasing number of symptomatic non-unions following conservative treatment of displaced fractures led to the development of various surgical approaches. Aim of this study was to evaluate the clinical and radiological outcome following operative treatment of displaced medial end clavicle fractures.

**Methods:**

Patients who presented with a displaced fracture of the medial clavicle between September 2012 and December 2019 were retrospectively enrolled in this study. All patients were operatively treated with open reduction and internal fixation (ORIF) using an anatomically precontoured locking compression plate (LCP) originally designed for the lateral clavicle (Synthes®, Umkirch, Germany). Functional outcome was recorded using the American Shoulder and Elbow Surgeons (ASES) Score, the Munich Shoulder Questionnaire (MSQ), Shoulder Pain and Disability Index (SPADI) and Constant Score.

**Results:**

Overall 18 patients with a mean age of 54.5 ± 23.5 years suffering from a displaced fracture of the medial clavicle were identified. The mean follow-up was 40.9 ± 26.2 months. The mean ASES accounted for 88.3 ± 20.8 points, the mean MSQ was 83.1 ± 21.7 points, the mean SPADI was 85.6 ± 22.5 and a mean normative age- and sex-specific Constant Score of 77.5 ± 19.1 points resulted. No minor or major complications were observed. Radiologic fracture consolidation was achieved in all patients after a mean of 6.4 months.

**Conclusion:**

Surgical treatment of displaced medial clavicle fractures using an anatomically precontoured locking plate originally designed for the lateral clavicle led to very good to excellent clinical and functional results.

**Trial registration:**

No: DRKS00024813, retrospectively registered 19.03.2021 (www.drks.de).

## Background

The incidence of medial clavicle fractures is far less frequent compared to midshaft or lateral clavicle fractures accounting for 2-9.3% of all clavicle fractures [2, 3, 23, 28, 35]. High-energy trauma (i.e. car or motorcycle accidents) represent the most common injury mechanism resulting in medial clavicle fractures [4, 26, 28]. Concludingly the majority of these patients are at high risk for concomitant injuries such as chest trauma or fractures of the shoulder girdle [3, 21, 23]. In this context, Throckmorton et al. reported that 90% of the patients with medial clavicle fractures sustained multiple injuries and were considered as “multitrauma patients” [28].

The common literature reveals a large number of publications regarding the treatment of midshaft as well as lateral clavicle fractures, however, little is known about optimal treatment of medial clavicle fractures [5, 9, 22, 23, 30]. Although several classification systems have been developed to describe the fracture pattern and the degree of dislocation, subsequent treatment guidelines are still missing [21, 28, 31]. In the past, non-surgical treatment has been the treatment of choice even in displaced medial clavicle fractures due to good healing rates [23]. In contrast, surgical treatment was considered in open fractures or relevant concomitant injuries concerning the shoulder girdle as well as injuries of neurovascular structures [4, 22]. However, symptomatic non-union leading to dysfunction occurred in 2.9 - 8% of the patients, therefore treatment strategy especially in physically active, but also young patients changed over the last years [4, 10, 23]. In addition, clavicle shortening following non-surgical treatment results in decreased moment generating- as well as total force generating capacity of the shoulder girdle muscles [18]. Therefore various surgical approaches using different fixation techniques (i.e. intramedullary implants, cerclage techniques, locking plate fixation) were used resulting in good to excellent results [7, 10, 13, 23, 29, 37].

Accordingly the aim of this study was to evaluate the clinical and radiological outcome using an anatomically precontoured locking plate originally designed for the lateral clavicle after a follow-up of at least one year.

## Methods

### Patients

Institutional review board approval was obtained prior to study begin by the local ethics committee. Patients who were operatively treated with an anatomically precontoured locking compression plate (Synthes®, Umkirch, Germany) for displaced medial clavicle fractures at our level 1 trauma center between September 2012 and December 2019 were retrospectively identified. The plate design was originally developed for the lateral clavicle third however by rotating the LCP by 180° the plate was perfectly adjusted to the medial clavicle third as depicted by our study group in 2014 [27]. All fractures were classified according to the AO/OTA, Edinburgh, Throckmorton&Kuhn and vanTongel (anatomically based, AB) classification [15, 21, 28, 31] (Table 1). Preoperative radiographs were performed in 2 planes (anterior-posterior perpendicular to cassette and anterior-posterior 30 degree angle cephalad). Additional computed tomography was performed in polytraumatized patients as well as in cases when conventional radiographs were not sufficient to adequately evaluate fracture morphology. Trauma mechanism, concomitant injuries as well as surgical duration and complications were recorded.

**Table 1:** Fracture Classification of dislocated medial end clavicle fractures. (AO/OTA [15], Edinburgh Classification [21], Throckmorton&Kuhn Classification including Displacement (minimal = < 2 mm, moderate 2-10 mm, severe > 10 mm) [28] and anatomically based (AB) Classification [31]

Patient No.	AO / OTA Classification	Edinburgh Classification	Throckmorton&Kuhn	Displacement	Anatomically Based
1	15 1.A	1B1	C	severe	1B
2	15 1.A	1B1	C	moderate	1B
3	15 1.A	1B1	A	moderate	1B
4	15 1.A	1B1	A	moderate	1B
5	15 1.A	1B1	C	severe	1B
6	15 1.C	1B2	D	moderate	1B
7	15 1.A	1B1	A	moderate	1B
8	15 1.A	1B1	C	moderate	1B
9	15 1.A	1B1	A	moderate	1B
10	15 1.A	1B1	A	minimal	1B
11	15 1.A	1B1	D	severe	1B
12	15 1.A	1B1	C	severe	1B
13	15 1.A	1B1	C	severe	1B
14	15.2A	1B2	A	moderate	1B
15	15.3A	1B2	C	moderate	1B

### Surgical technique and rehabilitation

The here presented surgical technique has been described by Siebenlist et al. [27]. Patients were placed in a beach-chair position with the affected arm in a mobile position. A longitudinal skin incision was made upon the medial clavicle with extension to the sternoclavicular (SC) joint without affecting it. After sharp dissection of the periosteum and debridement, the fracture was sparingly exposed. The position was checked clinically as well as via fluoroscopy. The plate was placed onto the clavicular shaft close to the SC joint without harming it.

In cases of multiple trauma patients additional surgery was performed (1) either immediately in our emergency department, (2) during plate fixation of the medial clavicle fracture or (3) after treatment and monitoring at the intensive care unit (see Table 2).

**Table 2:** Concomitant injuries and additional surgical treatment ((1) immediately in our emergency department, (2) during plate fixation of the medial clavicle fracture (fx) or (3) after treatment and monitoring in the intensive care unit)

Pat. No	Concomitant injury	Additional surgical treatment	Timing of surgery
1	–	–	–
2	AC-Joint dislocation	AC-Joint stabilisation	2
3	Humeral head fx (Neer IV.2), Subdural hemorrhage	Locking plate fixation of the humeral head	2
		External ventricular drainage	2
4	–	–	–
5	–	–	–
6	Pneumothorax, serial rib fx, discoligamentary instability C3/4	ACDF C 3/4 + plate fixation C3/4	3
7	Serial rib fx, Pneumothorax	Chest tube	1
8	–	–	–
9	Subarachnoid hemorrhage	Conservative management of SAH	1
	Pneumothorax	Chest tube	2
	Serial Rib fx	Locking plate fixation of the humeral head	2
	Floating Shoulder (med + lat. Clavicle Fx, multifragm. Scapula Fx)	Double Plate fixation of the clavicle (LCP)	
	prox. Humeral Fx		
10	Basilar Skull Fx	Plate fixation scapula	2
	Serial Rib Fx		
	Scapula Fx		
11	–	–	–
12	Traumatic brain injury 1°	Chest tube	1
	Serial Rib Fx		
	Pubic Fx		
	Pneumothorax		
13	Traumatic Brain injury 1°	Conservative management of SAH	3
	Basilar Skull Fx	Ankle: Plate / Screw osteosynthesis	
	Subarachnoid Hemorrhage		
	Trimalleolar Ankle Fx		
14	–	–	–
15	–	–	–

Postoperatively, immobilization was performed in an arm-sling (MediSling, Bayreuth, Germany) and patients started routine physical therapy on the first postoperative day. Abduction and flexion were restricted to 90° for the first six weeks. Return to sportive activity was allowed 6 weeks postoperatively.

### Follow-up

Shoulder function and pain as well as radiographic outcome were assessed at 6, 12, 26 weeks and one year after surgery. Implant removal was performed in case of irritation and explicit patients’ request. Functional outcome was recorded using the American Shoulder and Elbow Surgeons (ASES) Score [16] and the Munich Shoulder Questionnaire (MSQ) [24] allowing for self-assessment of the Shoulder Pain and Disability Index (SPADI) [19] and the sex and gender adapted Constant Score [6].

### Statistics

Data is given in terms of the arithmetic mean ± standard deviation. Descriptive statistics were used to illustrate the results. Statistical evaluation was performed using SPSS 25.0 (IBM Corp. Released 2017. IBM SPSS Statistics for Windows, Version 25.0. Armonk, NY: IBM Corp.).

## Results

**Fig. 1:**
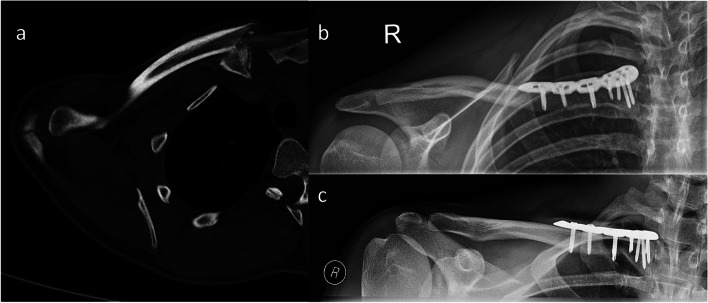
**a** Axial Computed tomography (CT) image of a medial end clavicle fracture preoperatively. **b** and **c** present x-rays in two planes after surgical fixation of the fracture using an anatomically preformed locking compression plate (LCP, Synthes, Umkirch, Germany)

Between September 2012 and December 2019 18 patients with a mean age of 54.5 ± 23.5 years were operatively treated for a displaced medial clavicle fracture using an anatomically precontoured locking compression plate (Fig. 1). One patient declined to participate in this study. Two patients moved so that follow up examinations were not performed. These three patients were excluded from this study. Overall 15 patients (12 male, 3 female) with a mean age of 50.5 ± 24.4 years were available for all postoperative follow ups and thus participated in this study. Mean follow up was 40.9 ± 26.2 months with a minimum follow up of 12 months (range 12.6 - 83.2 months). Concomitant injuries were found in 8/15 patients (53.3%; see Table 2). Surgical treatment for concomitant injuries was necessary in 7/15 patients (46.6%). Thoracic injuries (i.e. pneumothorax) were treated immediately in our emergency department. Injuries of the ipsilateral upper extremity (proximal humerus fracture (*n* = 2, 13.3%), AC-joint dislocation (*n* = 1, 6,6%)), scapular fracture (n = 1, 6,6%) were surgically treated in the same session (*n* = 4, 26.6%) as the medial clavicle fracture (Table 2). The functional outcome of these 4 patients was excluded to avoid misinterpretation of a multiple injured upper extremity. Exclusion of these 4 patients did not statistical significant alter the overall functional outcome (*p* = .806). Surgical treatment of a vertebral body fracture (cervical spine) was performed with a certain delay after surgery of the medial clavicle fracture (*n* = 1, 6.6%). Non-surgical, conservative treatment was performed in patients suffering from concomitant injuries such as rib fractures (*n* = 5) and one non-displaced pubic fracture (n = 1).

Neither minor (wound-healing disorders etc.) nor major (non-union, re-fracture, revision etc.) complications were observed during follow-up examinations in our outpatient clinic for medial clavicle fractures. Overall 7 implant removals were performed due to irritation and patients’ request after a mean of 22.2 ± 8.0 months. No re-fractures were observed until last follow up examination. Bone healing was found on conventional radiographs performed at the follow-up examinations in all patients after a mean of 6.4 ± 3.9 months.

The mean ASES accounted for 88.3 ± 20.8 points, the mean normative age- and sex-specific Constant Score was 77.5 ± 19.1 points, the mean SPADI 85.6 ± 22.5 and the mean MSQ resulted in 83.1 ± 21.7 points. 6 patients returned to their preoperative activity / sports level with only minor restrictions in overhead activities, 9 patients did not perform any sports prior to surgery. All preoperative sportive activities could be carried out postoperative. Overall mean time for return to sports for riding bicycle, fitness, jogging, soccer and snowboarding was 8.4 ± 3.4 months.

## Discussion

Operative treatment of medial clavicle fractures utilizing a 180° twisted locking compression plate (invented for the lateral clavicle) leads to good to excellent results. Not only the degree of dislocation but also patients’ activity level are important factors for decision making whether to operate or treat the fracture conservatively. The incidence of medial clavicle fractures is rather low especially when compared to midshaft or lateral clavicle fractures accounting for 2-9.3% of all clavicle fractures [2, 3, 23, 28, 35]. Therefore we reached out to address this rare entity and elucidate findings including concomitant injuries and present results to our operative techniques as described by Siebenlist et al. [27].

Treatment of medial clavicle fractures is still challenging and controversially discussed in the upper extremity community. A certain consensus exists for treating non-displaced fractures conservatively. In this context several authors prefer open reduction and internal fixation of displaced fractures in physically active patients [7, 12, 29]. The use of an anatomically precontoured locking plate showed promising functional results in small case studies [7, 27, 34].

Current classification systems allow for a description of fracture pattern and degree of dislocation of medial clavicle fracture, however distinct treatment guidelines are still missing. Therefore van Tongel et al. developed an anatomically based classification system focusing on the fracture dislocation (bony vs no bony contact of fragments) and the location of the fracture line in relation to the costo-clavicular ligament and the SC joint capsule [31]. A worse functional outcome (Constant Score 70 points, SD 14) and a higher incidence of symptomatic non-unions (4/13) following nonoperative treatment was associated with a lack of bony contact in fractures medial of the costoclavicular ligament (Typ 1B) compared to fractures with bony contact of the fragments (Typ 1A, Constant Score 79 points, SD 14; no non-union). In the current study all fractures were classified as Typ 1B according to van Tongel et al. [31]. Surgical treatment resulted in a considerable higher Constant Score of 77.6 ± 17.4 points and bony union in all cases compared to the study group of van Tongel et al. Therefore our results, in accordance with those of van Tongel et al., state the prognostic outcome for conservative treatment of Typ 1B fractures inferior. Therefore primary operative treatment should be recommended in physically active patients.

The presented study presents a retrospective trial on the clinical as well as radiological results of 15 patients suffering from dislocated fractures of the medial clavicle treated with the just mentioned anatomically precontoured locking plate originally designed for lateral clavicle fractures. The mean age of the enrolled 15 patients was 50.5 ± 24.4 years with a male-female ratio of 12:3. Unfortunately, the small cohort size is a well-known problem in outcome evaluation studies of pathologies with a low incidence such as medial clavicle fractures. Therefore, the strength of the obtained results can only considered as the fundament as well as good starting point for further analysis. However, in this context the recent literature also only provides studies enrolling little patient numbers or even lower compared to the presented study. Therefore we contribute to the poor data situation in the recent literature of this low-incidence-disease with a study of a comparatively high number of patients addressing concomitant injuries including all current classification systems.

Various surgical techniques and different implants have been in use for open reduction and internal fixation of medial end clavicle fractures [1]. None of the available implants has been especially developed for the anatomy of the medial clavicle and a standardized surgical approach has not yet been established. Due to the short metaphyseal fracture fragment, Li et al. suggested to use a reconstruction plate as a bridging plate technique across the sternum to the contralateral healthy clavicle in terms of a temporary arthrodesis [11]. The authors further report bony union and good clinical outcome with high satisfaction 6 months postoperatively (DASH 23.33). However, for this across-sternum technique two incisions with related crucial soft tissue trauma are necessary and both non-affected SC joints are impaired so that implant removal is mandatory. However, this is a newly described technique (case report) and long term follow up is missing why general conclusions should be omitted. An alternative to gain sufficient fixation of small metaphyseal fragments as a common problem also in fractures of the lateral clavicle could be achieved by the use of locking compression plates. Depending on the type of implant the lateral extension allows for the placement of 2.3 - 2.7 mm divergent locking screws increasing “pull-out” - strength.

To avoid bending of locking plates resulting in decreased stability, anatomically precontoured locking plates were developed for numerous locations of the human skeleton presenting with a high incidence of injuries. However, the clavicle presents with a large anatomic variability in its sigmoid shape. Vancleef et al. calculated a statistical shape model of the clavicle reported data on the average clavicular geometry [32]. The authors concluded that several plate shapes are needed to fit all types of clavicle fractures.

Previously, Titchener et al. used a precontoured locking plate originally designed for the lateral clavicle for fixation of medial clavicle fractures [29]. In all cases the plate was helical bended in a 90° fashion around its axis to fit to the medial clavicle. All fractures healed properly and no implant failure was reported. However, bending of locking plates can reduce the stiffness of the implant with an increased risk of implant failure [14, 20].

In contrast to Titchener et al., as reported, the lateral LCP perfectly matches the anatomy of the medial clavicle without additional bending needed. Frima et al. reported on using a VA (various angle)-LCP (distal humerus) with additional lateral support as “well-fitting” with excellent functional outcome which is in accordance to our findings [7, 12, 27].

Hardware removal due to soft tissue irritation is a common problem in plate fixation of clavicle fractures and was performed in 7 / 15 cases in the presented study. These results are comparable to the results of Frima et al. in 2018 [7]. Routine removal of implants remains controversially discussed with a lack of evidence based guidelines. In a recent study our study group reported on improved functional outcome and increased activity levels after implant removal [17, 36]. However, until now, the german-speaking society of Traumatology (DGU) appreciates implant removal as no mandatory procedure [25]. Due to typical surgical risks and complications, implant removal should only be performed in symptomatic patients (i.e. wearing heavy bags or due to weather changes) [33]. Removal of implants is most commonly performed in the clavicle. Various reason have been identified (i.e. irritation, pain etc). Due to a prominent subcutaneous position of the implant especially in patients with poor soft tissue coverage irritation rates in this area are higher compared to other regions [8]. In this context Titchener et al. positioned in all 8 enrolled patients the plate over the anterior surface of the medial clavicle and superior on the shaft so that no implant removal was necessary. However, two patients of his patients’ cohort reported a slight prominent feeling of the plate [29].

### Limitations

There are several limitations to be considered when interpreting the presented results. First, the retrospective character of the data analysis of our in-house fracture register may be inaccurate and may not provide the quality of a prospective data selection. Secondly, we did not compare conservatively treated patients to operatively treated ones yet with respect to degree of fracture dislocation this comparison would not have been appropriate. Future investigations with prospective randomized comparisons of operative treatment in medial end clavicle fractures need to be performed and is focus of ongoing research of our study group.

## Conclusion

Locking compression plate fixation (originally developed for the lateral clavicle) of displaced medial end clavicle fractures provides decent stability due to diverging screws in the medial fracture portion in physically active patients including a very good to excellent functional outcome.

## Data Availability

The datasets used and analyzed during the current study are available from the corresponding author on reasonable request.

## Abbreviations

AO: OTA Arbeitsgemeinschaft für Osteosynthesefragen Orthopaedic Trauma Association
DASH: Disability of Arm, Shoulder and Hand
DGU: Deutsche Gesellschaft für Unfallchirurgie
LCP: Locking compression plate
MSQ: Munich Shoulder Questionnaire
ORIF: Open reduction – internal fixation
SPADI: Shoulder Pain and Disability Index
